# The Functions of the A1A2A3 Domains in Von Willebrand Factor Include Multimerin 1 Binding

**DOI:** 10.1160/TH15-09-0700

**Published:** 2016-04-07

**Authors:** D’Andra N. Parker, Subia Tasneem, Richard W. Farndale, Dominique Bihan, J. Evan Sadler, Silvie Sebastian, Philip G. De Groot, Catherine P. M. Hayward

**Affiliations:** 1 Pathology and Molecular Medicine, McMaster University, Hamilton, Ontario, Canada; 2 Department of Biochemistry, University of Cambridge, Cambridge, UK; 3 Department of Medicine, Washington University School of Medicine, Saint Louis, Missouri, USA; 4 Laboratory of Clinical Chemistry and Haematology, University Medical Centre Utrecht, Utrecht, Netherlands; 5 Medicine, Pathology and Molecular Medicine, McMaster University, Hamilton Regional Laboratory Medicine Program, Hamilton, Ontario, Canada

**Keywords:** von Willebrand factor, platelet glycoproteins, adhesion molecules, collagens

## Abstract

Multimerin 1 (MMRN1) is a massive, homopolymeric protein that is stored in platelets and endothelial cells for activation-induced release. *In vitro*, MMRN1 binds to the outer surfaces of activated platelets and endothelial cells, the extracellular matrix (including collagen) and von Willebrand factor (VWF) to support platelet adhesive functions. VWF associates with MMRN1 at high shear, not static conditions, suggesting that shear exposes cryptic sites within VWF that support MMRN1 binding. Modified ELISA and surface plasmon resonance were used to study the structural features of VWF that support MMRN1 binding, and determine the affinities for VWF-MMRN1 binding. High shear microfluidic platelet adhesion assays determined the functional consequences for VWF-MMRN1 binding. VWF binding to MMRN1 was enhanced by shear exposure and ristocetin, and required VWF A1A2A3 region, specifically the A1 and A3 domains. VWF A1A2A3 bound to MMRN1 with a physiologically relevant binding affinity (K_D_: 2.0 ± 0.4 nM), whereas the individual VWF A1 (K_D_: 39.3 ± 7.7 nM) and A3 domains (K_D_: 229 ± 114 nM) bound to MMRN1 with lower affinities. VWF A1A2A3 was also sufficient to support the adhesion of resting platelets to MMRN1 at high shear, by a mechanism dependent on VWF-GPIbα binding. Our study provides new information on the molecular basis of MMRN1 binding to VWF, and its role in supporting platelet adhesion at high shear. We propose that at sites of vessel injury, MMRN1 that is released following activation of platelets and endothelial cells, binds to VWF A1A2A3 region to support platelet adhesion at arterial shear rates.

## Introduction

von Willebrand factor (VWF) is a massive, shear-sensitive, homopolymeric protein that is important for initiating platelet adhesion at high shear (1). VWF normally circulates in a globular form that unfolds, elongates and self-associates in response to high hydrodynamic shear (1–4). Shear-exposed VWF reveals multiple cryptic binding sites that support VWF binding to platelet glycoprotein (GP) Ibα (5–8) and platelet aggregation (9). The exposure of VWF to high shear also controls interactions with haemostatic ligands that modulate its platelet adhesive properties, including binding to the adhesive protein multimerin 1 (abbreviation: human: MMRN1, mouse: Mmrn1) (10). MMRN1 is a massive, variablysized, disulfide-linked, EMILIN homopolymeric protein that is proposed to support platelet function at sites of vessel injury (10–14).

MMRN1 and VWF have interesting similarities, although they are structurally distinct proteins (10, 11, 13). Both MMRN1 and VWF are expressed and stored by megakaryocytes, platelets and endothelial cells for stimulus-induced release, but unlike VWF, MMRN1 is not normally detectable in plasma (13, 15, 16). VWF and MMRN1, respectively, are the main binding proteins for the homologous coagulation factors factor VIII (17) and factor V (FV) (18), and similarly interact with the phospholipid binding domains of these cofactors to modulate coagulation (19, 20). Like VWF, MMRN1 has roles in supporting platelet adhesive functions (10, 14, 21). Mice with spontaneous deletion of the Mmrn1 and α-synuclein gene have both defective platelet adhesion and platelet-rich thrombus formation in arterioles injured with ferric chloride (14). Additionally, these mice have impaired platelet adhesion to collagen at a high shear rate (1500 s^-1^) that is corrected by added MMRN1 (14). Like VWF, MMRN1 binds to collagens types I and III, and VWF and MMRN1 together enhance platelet adhesion to collagen at a high shear rate (10). MMRN1 also supports platelet adhesion at low shear rates (e. g. 150 s^-1^) by mechanisms that require GPIbα but not VWF, whereas at a high shear rate, MMRN1 supports platelet adhesion by a mechanism that requires both GPIbα and VWF (10). VWF associates with MMRN1 under high shear rates, but not under static conditions (10), suggesting that high shear exposes cryptic sites within VWF that are involved in MMRN1 binding. MMRN1 does not detectably bind to GPIbα (10), suggesting that VWF binding to MMRN1 and to GPIbα supports platelet adhesion to MMRN1 at high shear. Nonetheless, the mechanism underlying VWF interactions with MMRN1 has not been directly evaluated.

The shear-sensitive A1A2A3 region of VWF is important for platelet adhesion because this region contains the binding sites for GPIbα (7, 22), subendothelial collagens (types I, III, IV, and VI) (23–27), and the plasma protease ADAMTS13 (a disintegrin and metalloproteinase with a thrombospondin type 1 motif, member 13) (28). In response to vascular injury, the structurally homologous VWF A1 and A3 domains bind to subendothelial collagens (23–27), and this association enhances exposure of the cryptic A1 domain binding site for GPIbα (1, 4, 8, 29, 30). VWF A1 domain also possesses binding sites for non-physiological modulators of VWF-GPIbα binding, such as ristocetin, that also expose cryptic sites within VWF A1 domain (22, 31). VWF A2 domain possesses unique structural features that regulate its response to high shear and proteolysis by ADAMTS13, which downregulates the plateletadhesive properties of VWF by cleaving it into smaller polymers (1, 28, 32, 33). Like full-length VWF, VWF A1A2A3 domains respond to high shear (6, 34, 35) and support platelet adhesion at high shear by GPIbα-dependent mechanisms (6, 34, 36). As VWF A1A2A3 domains have been used to probe the mechanism and affinity of VWF binding to other proteins (34, 36, 37), we postulated that these domains would be helpful to investigate the mechanism of VWF-MMRN1 binding.

The apparent shear-dependent binding of VWF to MMRN1 led us to postulate that the shear-sensitive A1A2A3 region of VWF (which is critical for GPIbα-dependent platelet adhesion [7, 22]) is required for MMRN1 binding and platelet adhesion to MMRN1 at high shear. We tested these possibilities using domain-deleted VWF mutant proteins lacking one or more VWF A domains, and monomeric VWF mutant proteins possessing one or more A domain(s).

## Materials and methods

This study was performed with institutional ethics review board approval, in accordance with the revised Helsinki Declaration on human research. For platelet adhesion experiments, blood was collected with informed consent from two or three healthy individuals and from an individual with type 3 von Willebrand disease (VWD) with undetectable plasma and platelet VWF.

### Protein preparation and sources

Affinity purified recombinant MMRN1 was prepared as described (21) for protein binding and platelet adhesion assays. Biotin-labelled MMRN1 was prepared and immobilised as described (20) to assess the binding affinities of VWF A domains for MMRN1 by surface plasmon resonance (SPR). Bovine serum albumin (BSA; Sigma-Aldrich Canada, Oakville, ON, Canada) was used as the negative control for binding and adhesion assays.

VWF mutant proteins tested for MMRN1 binding included: full-length wild-type (WT)-VWF; VWF lacking the A1A2A3 domains (ΔA1A2A3) (38); and VWF lacking individual A domains (ΔA1, ΔA2, A A3) (22, 23, 28). The concentrations of VWF mutant proteins were determined by an enzyme-linked immunosorbent assay (ELISA), as described (22), using a horseradish peroxidase conjugated, rabbit polyclonal antibody (P0226, Dako, Glostrup, Denmark) to detect VWF and mutant VWF proteins lacking one or more A domains.

Monomeric VWF mutant proteins tested for MMRN1 binding included: A1A2A3, A1, A2, and A3 (A2 domain was provided by Dr. E. Huizinga) (39–42). Concentrations of these mutant proteins were determined by bicinchoninic acid (BCA) assay (Thermo Scientific, Waltham, MA, USA).

Triple-helical type III collagen peptides, tested as inhibitors of MMRN1-VWF binding included: the collagen peptides (III-23: GPC-(GPP)_5_-GPOGPSGPRGQOGVMGFOGPKGNDGAO- (GPP)_5_-GPC-NH_2_ and a smaller peptide designated GPRGQOGVMGFO, of full sequence GPC- (GPP)_5_-GPRGQOGVMGFO-(GPP)_5_-GPC-NH_2_ that specifically bind to VWF A3 domain (43). The control peptide (GPP: GPC- (GPP)10-GPC-NH_2_), that does not bind VWF (43), served as a negative control. All of these collagen peptides were verified not to bind to MMRN1 (data not shown).

### VWF-MMRN1 binding assays

Modified ELISA (10) were used to assess the ability of immobilised MMRN1 (0.5 μg per well) to support the binding of VWF (0–0.2 μg per well) that was untreated, pre-treated with ristocetin (1 mg/ ml), or exposed to shear on a mini vortex mixer for 20–30 seconds (s) (2500–3200 rpm; VWR, Radnor, PA, USA) as described (44). For other assays, the binding of VWF A1A2A3 (0–0.3 μg per well) to MMRN1 was assessed. For some experiments, VWF binding to MMRN1 was assessed with or without simultaneous addition of the VWF binding collagen peptides III-23, GPRGQOGVMGFO, or control peptide GPP (each peptide was tested at 250 μg/ml, without preincubation with VWF). For other experiments, VWF binding to MMRN1 was assessed with or without simultaneous addition of 2 μg/ml control mouse immunoglobulin G (mIgG; Jackson ImmunoResearch, West Grove, PA, USA) or 2 μg/ml of an inhibitory monoclonal antibody against VWF A1 (MCA4683; AbD Serotec, Raleigh, NC, USA) or A3 domain (RU5) (43); both inhibitory antibodies were assessed at a concentration that inhibits VWF binding to GPIbα and collagen, respectively (43).

For binding assays, VWF A1A2A3 mutant proteins were diluted in HEPES-Tyrode buffer (137 mM NaCl, 2 mM KCl, 0.3 mM NaH_2_PO_4_, 1 mM MgCl_2_, 5 mM HEPES, 12 mM NaHCO_3_, pH 7.4), supplemented with 2 mM CaCl_2_. VWF mutant proteins bound to MMRN1 were detected using P0226 and the chromogenic substrate tetramethylbenzidine (TMB) (ALerCHEK, Inc. ColorburstTM Blue, Portland, ME, USA); reactions were stopped by adding 1 M H_2_SO_4_, and relative binding was reported as optical density (OD) measured at 450 nm (10). Binding assays were done three times with each sample, tested in duplicate or triplicate.

SPR was performed to assess the binding affinity of VWF A1A2A3 for MMRN1 and the binding of individual A domains to MMRN1 because ELISA using P0226 showed inconsistent binding of these domains to immobilised MMRN1. Real-time association and dissociation data were obtained using BIAcoreX instrument (BIAcore, Uppsala, Sweden). For the evaluation of VWF A1A2A3 binding to MMRN1, SPR experiments were conducted using different contact times (2–10 minutes [min]) and injection flow rates (10–40 μ/min) to avoid mass transfer effects.

For kinetic estimates, VWF A domains (in HEPES-Tyrode running buffer, with 0.005 % P-20) were perfused over biotin-labelled MMRN1 (immobilised on streptavidin-coated sensor chips). VWF mutant proteins were tested at concentrations of 0–2000 nM, and those that are known to self-aggregate at concentrations above 500 nM (49) were tested at lower concentrations (≤ 250 nM for A1, ≤ 25 nM for A1A2A3). The baseline surface was restored by 1 M NaCl containing 50 mM NaOH, as described (20). Binding affinities were determined using BIAEvaluation software (version 4.1) and best-fit models for data (determined by visual inspection and Chi^2^ values), as described (20). For VWF A1A2A3 and VWF A1, the two state conformational model was used: K_D_ = (k_d1_·k_d2_)/ k_a1_(k_d2_+k_a2_). For VWF A3, the 1:1 Langmuir binding model was used: K_D_ = k_d_/k_a_ (20). SPR experiments were repeated two or more times for each analyte, using different MMRN1 preparations, with immobilization levels ranging from 500–1000 resonance units (RU).

### Platelet preparation and microfluidic platelet adhesion assays

Blood was collected into low-molecular-weight heparin anticoagulant (final: 20 U/ml dalteparin sodium, Pfizer Canada Inc., Mississauga, ON, Canada) to prepare calcein-labelled, washed platelets for adhesion assays, as described (10). After collecting platelet-rich plasma, platelets were incubated with calcein acetoxymethyl ester (2.5 µg/ml; Invitrogen Canada Inc., Burlington, ON, Canada), followed by washing and resuspension in HEPES-Tyrode buffer with added red cells (final: 300 × 10^6^ platelets/ml; haematocrit 45 %) (10).

High shear (1500s^-1^) platelet adhesion assays were performed to determine if VWF A1A2A3 supported platelet adhesion to MMRN1. Briefly, Vena8Fluoro+ Biochips (Cellix, Dublin, Ireland) were coated with MMRN1 or BSA (50 µg/ml overnight, 4 °C), then blocked with 2 % BSA in HEPES-Tyrode buffer for 2 hours at room temperatures before use. Protein-coated chambers were then pre-treated with VWF A1A2A3 (40 nM, estimated based on a 72.5 kDa monomer size), WT-VWF (40 nM, positive control; estimated based on a 250 kDa monomer size), or 0.2 % BSA (negative control) in HEPES-Tyrode buffer, by flowing these proteins over the surfaces at high shear (1500 s^-1^, 6 min) using Mirus™ 2.0 Nanopump (Cellix, Dublin, Ireland) and VenaFluxAssay™ Software (Cellix, Dublin, Ireland). Next, labelled platelets in reconstituted blood were drawn through the chambers at high shear (1500 s^-1^, 3 min), followed by a wash step (0.2 % BSA in HEPES-Tyrode supplemented with 1 U/ml heparin) before endpoint analysis of adherent platelets (% area covered by platelets) by microscopy, as described (10). For some experiments, MMRN1-coated chambers were perfused with WT-VWF, with or without preincubation (15 min, 37^o^C) with 20 µg/ml of control mouse IgG or MCA4683 (inhibitory antibody against VWF A1). Platelet adhesion assays were conducted at least two times with different protein preparations.

### Statistical analysis

Data were reported as mean (minus background) ± standard error of the mean (SEM) unless otherwise stated. Comparative data with two groups were evaluated by an unpaired two-tailed Student’s t-test. Comparative data with three or more groups were evaluated by one-way analysis of variance (ANOVA) followed by Tukey multiple comparisons post-hoc test (GraphPad Prism 5, La Jolla, CA, USA). p values < 0.05 were considered statistically significant.

## Results

### Static binding of WT-VWF and VWF A domains mutant proteins to MMRN1

**Figure 1:**
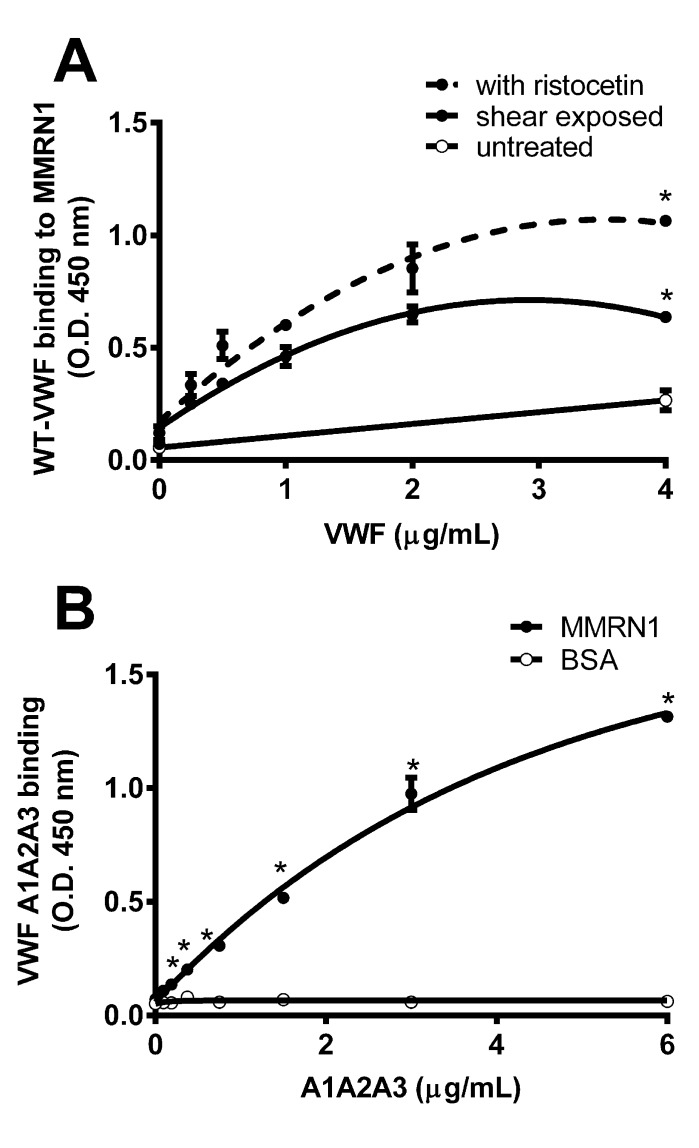
**VWF-MMRN1 binding, in modified ELISA.** A) Binding of untreated (open circles, solid line), shear-exposed (closed circles, solid line) and ristocetin-treated (closed circles, dashed line) WT-VWF to immobilised MMRN1. * indicates different (p < 0.05) from untreated VWF. B) Binding of VWF A1A2A3 to immobilised MMRN1 (closed circles) and the negative control protein BSA (open circles). * indicates different (p < 0.01) from BSA. Binding data are representative of 2–3 separate experiments, expressed as the mean OD 450 nm ± SD.

In modified ELISA, WT-VWF bound to immobilised MMRN1 in a concentration-dependent and saturable manner when exposed to ristocetin or shear (▶ Figure 1A). The binding of ristocetin and shear exposed WT-VWF exceeded the binding of untreated VWF to MMRN1 (p < 0.05, ▶ Figure 1A), which was not detectable in some experiments. VWF A1A2A3 (tested without exposure to shear or ristocetin) also showed saturable, concentration-dependent binding to MMRN1 in modified ELISA (p < 0.01 relative to BSA; ▶ Figure 1B).

**Figure 2:**
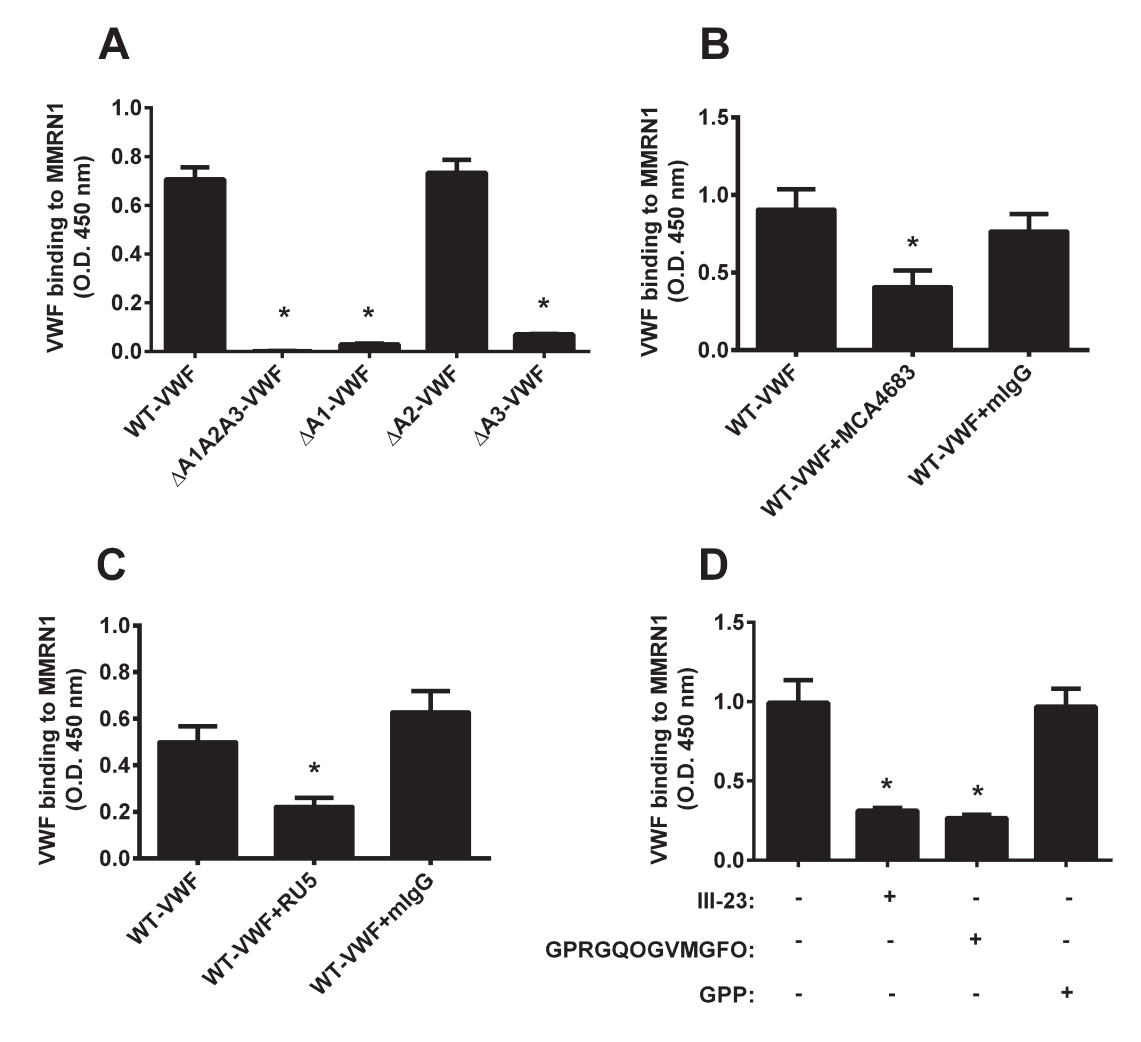
**Binding of WT-VWF and VWF mutant proteins to immobilized MMRN1, with or without inhibitors.** A) The binding of 0.5 µg/ml shear-exposed WT-VWF, and VWF mutant proteins lacking all or one A domain (ΔA1A2A3, ΔA1, ΔA2, ΔA3), to MMRN1. * indicate reduced binding (p < 0.01), relative to WT-VWF. B) Effect of MCA4683 (2 µg/ml, a VWF A1 domain antibody that inhibits GPIbα binding) or negative control mIgG (2 µg/ml) on VWF-MMRN1 binding in the presence of ristocetin. * indicates reduced (p < 0.05), relative to WT-VWF with no inhibitor. C) Effect of RU5 (2 µg/ml, a VWF A3 domain antibody that inhibits collagen binding) and mIgG (2 µg/ml; negative control) on VWF-MMRN1 binding. * indicates reduced (p < 0.05), relative to WT-VWF with no inhibitor. D) Effect of type III collagen peptides that bind to VWF A3 domain (III-23: GPOGPSGPRGQOGVMGFOGPKGNDGAO, and GPRGQOGVMGFO) and the negative control peptide, GPP, on VWF-MMRN1 binding. * indicates reduced, (p < 0.05), relative to WT-VWF with no inhibitor. WT-VWF binding (1 µg/ml) to immobilised MMRN1 was measured following simultaneous addition of VWF and an antibody or peptide. Panels show representative (D) or pooled (A-C) data for three separate independent experiments, expressed as the mean OD 450 nm ± SEM.

The contributions of VWF A domains to MMRN1 binding was further evaluated with shear exposed, domain deleted VWF mutant proteins. Compared to WT-VWF, VWF mutant proteins lacking all A domains (ΔA1A2A3) showed impaired binding to MMRN1 (p < 0.01), as did VWF mutant proteins lacking the A1 (ΔA1) or A3 (ΔA3) domains (p < 0.01), but not the VWF mutant lacking the A2 domain (ΔA2; ▶ Figure 2A). ΔA1A2A3, ΔA1, and ΔA3 VWF mutant proteins had binding that was reduced to the level of background (▶ Figure 2A). WT-VWF binding to immobilised MMRN1 in modified ELISA was reduced by monoclonal antibodies that inhibit the VWF A1 (~55 % reduction; MCA4683; p < 0.05, relative to VWF alone, ▶ Figure 2B) and A3 (~55 % reduction; RU5; p < 0.05, relative to VWF alone; ▶ Figure 2C) domains, and by the type III collagen peptides III-23 and smaller peptide, GPRGQOGVMGFO (~70 % reduction; p < 0.01, relative to no added peptide; ▶ Figure 2D). Both collagen peptides that showed inhibitory effects are known to bind VWF A3 domain and inhibit VWF A3 binding to collagen type III at the concentration tested (43). The negative controls, collagen peptide GPP and mIgG, had no effect on WT-VWF binding to immobilised MMRN1 (▶ Figure 2B-D). The effect of collagen peptides on VWF-MMRN1 binding could not be evaluated by SPR because the peptides showed a high level of non-specific binding to the sensorchips.

### The effect of inhibitory antibody against VWF A1-GPIbα binding, on platelet adhesion to MMRN1 at high shear (1500 s^-1^)

**Figure 3:**
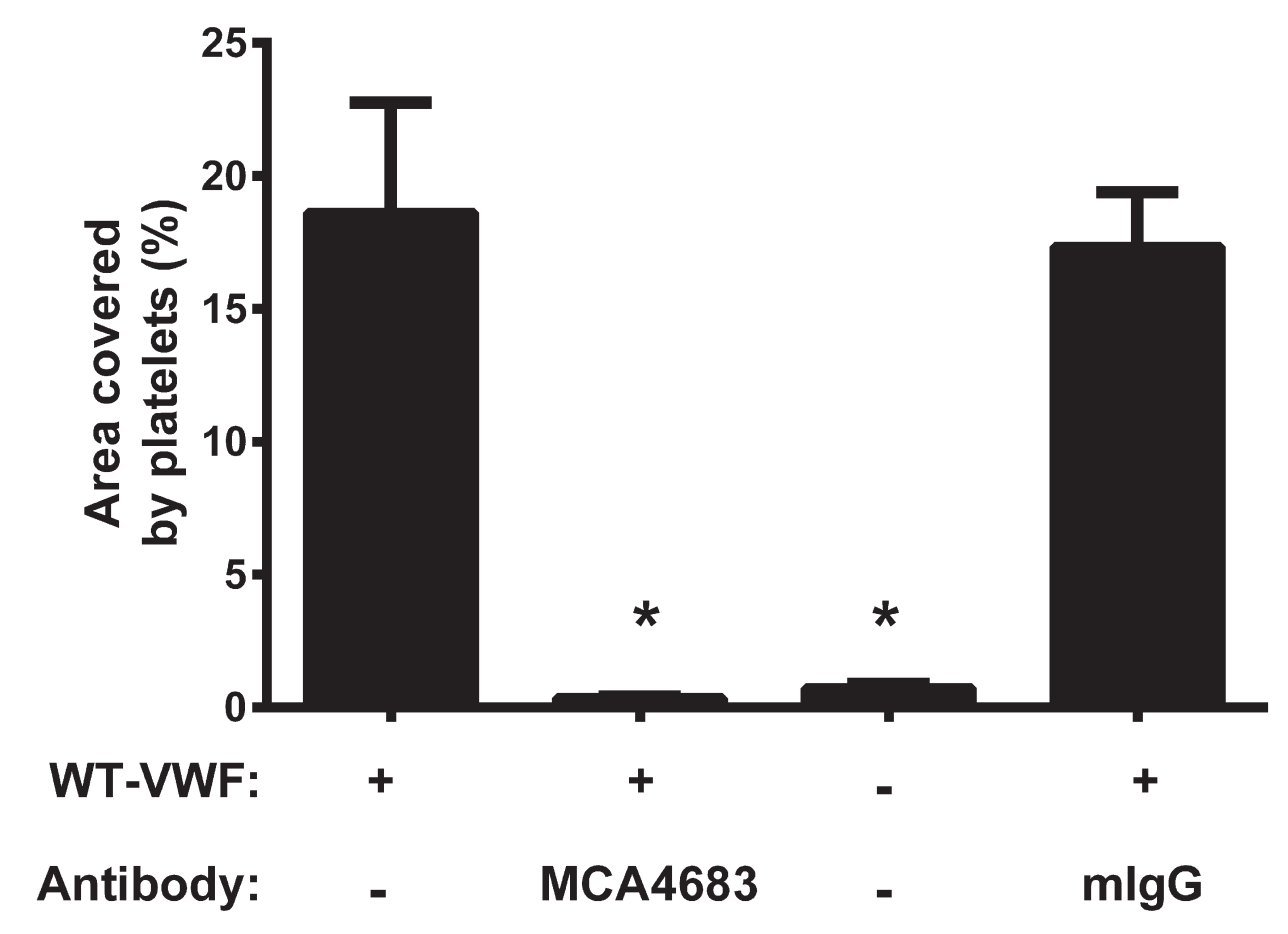
**Effect of inhibitory antibodies on the ability of VWF to support high shear platelet adhesion to immobilised MMRN1.** Bars show mean ± SEM for the percentage of the surface area [%] covered by platelets for the adhesion of control platelets (300 × 10^6^/ml) to immobilised MMRN1, tested at a shear rate of 1500 s^-1^ after pre-treatment with: WT-VWF (40 nM, positive control); WT-VWF + MCA4683 (20 µg/ml, inhibits VWF-GPIbα binding); BSA (negative control); or WT-VWF + mIgG (20 pg/ml, negative control). Reduced platelet adhesion to MMRN1 is indicated (*; p < 0.001, relative to MMRN1 pre-treated with WT-VWF only; data representative of two independent experiments).

MMRN1 surfaces treated with VWF supported the adhesion of resting control platelets better than MMRN1 surfaces pre-treated with BSA (p < 0.001) and this enhancing effect of VWF was abrogated by the inhibitory antibody MCA4683 that blocks VWF A1-GPIbα binding (p < 0.001 relative to WT-VWF alone) but not by control mIgG (▶ Figure 3).

### SPR analysis of the binding of VWF A domains to MMRN1

**Figure 4:**
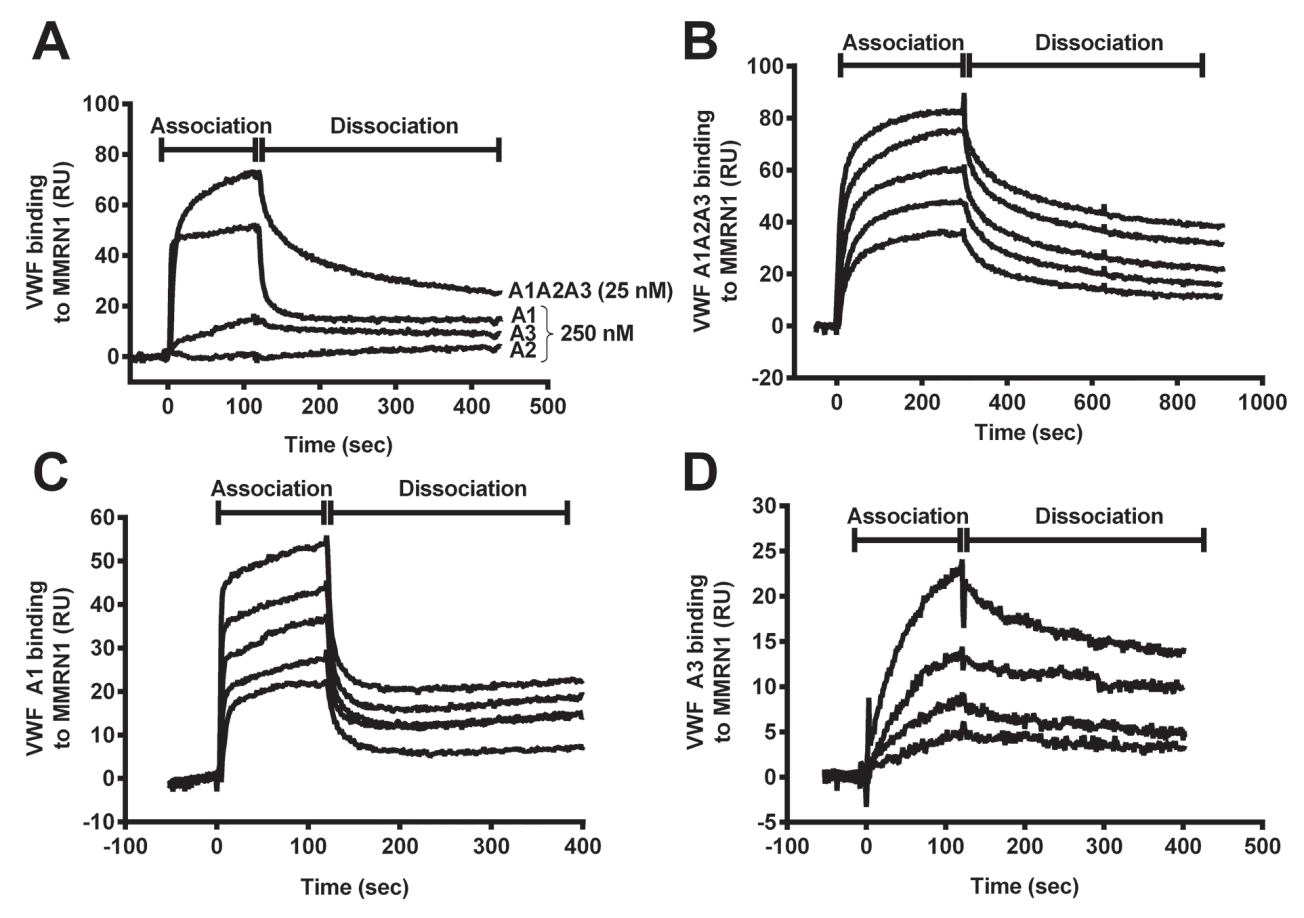
**Real-time association and dissociation curves for VWF A domains binding to MMRN1, evaluated by SPR.** A) Association and dissociation profiles for VWF: A1A2A3 (25 nM); and A1, A2, and A3 domain (250 nM) binding to MMRN1-coated sensor chips (~1000 RU immobilised MMRN1; flow rate: 20 μl/min). B, C and D) SPR sensorgrams for the real-time binding of different analytes to MMRN1-coated sensor chips (data for chips with ~1000 RU immobilised MMRN1 are shown): B) VWF A1A2A3 were tested at analyte concentrations (nM, top to bottom): 25, 18.8, 9.4, 4.7 and 3.1; C) VWF A1 domain was tested at analyte concentrations (nM, top to bottom) of: 250, 125, 62.5, 31.3 and 15.6; and D) VWF A3 domain was tested at analyte concentrations (nM, top to bottom) of: 2000, 1000, 500 and 250. All data shown was representative of 2–3 experiments.

SPR analysis indicated that VWF A1, A3, and A1A2A3 domains bound to MMRN1, unlike the VWF A2 domain (Figure 4A). VWF A3 domain showed slower association and dissociation from MMRN1 than the A1 domain (Figure 4A). VWF A1A2A3 binding to MMRN1 showed some features of VWF A1 domain and A3 domain binding to MMRN1 (Figure 4A). Like VWF A1 domain, VWF A1A2A3 showed rapid initial association to MMRN1, followed by a slower gradual association that was also observed with the VWF A3 domain (Figure 4A). Like VWF A1 domain, VWF A1A2A3 showed an initial rapid dissociation from MMRN1, that was followed by a second slower phase of dissociation that resembled dissociation of the VWF A3 domain from MMRN1 and the later phase of VWF A1 domain dissociation from MMRN1 (Figure 4A).

▶ Table 1 summarises the binding kinetic estimations for the real-time binding of VWF A1A2A3, A1, and A3 domains to MMRN1. In agreement with the association and dissociation profiles, the binding of VWF A1A2A3 and the A1 domain to MMRN1 showed best fit (Chi^2^: 2.32–9.34) to a two-state, conformational change binding model (A+L← → AL ← → AL*), where A (analyte: A1A2A3 or A1) and L (Ligand: MMRN1) form two stable complexes (AL and AL*). The binding of VWF A3 domain to MMRN1 showed best fit (Chi^2^: 1.07–3.19) to a simple 1:1 Langmuir binding model (A+L ← → AL), unlike VWF A1A2A3 (Chi^2^ ≥ 30).

Using the two-state conformational model, VWF A1A2A3 had a lower K_D_ (representative of higher apparent affinity) for MMRN1 (K_D_: 2.0 ± 0.4 × 10^–9^ M; ▶ Figure 4B) than the VWF A1 domain (K_D_: 39.3 ± 7.7 × 10^–9^ M, p < 0.01; ▶ Figure 4C). VWF A1A2A3 (k_a1_: 2.6 ± 0.9 × 10^6^ M^-1^s^-1^; k_a2_: 4.2 ± 0.3 × 10^–3^ s^-1^) and VWF A1 (k_a1_: 1.2 ± 0.8 × 10^6^ M^-1^s^-1^; k_a2_: 1.0 ± 0.5 × 10^–2^ s^-1^) had similar on-rates for binding MMRN1 (p = 0.35 and 0.28, respectively). VWF A1A2A3 had a slower initial off-rate (k_d1_: 2.7 ± 0.3 × 10^–2^ s^-1^) than the A1 domain (k_d1_: 1.3 ± 0.2 × 10^–1^ s^-1^, p < 0.01). A second slower phase of the dissociation was evident with both VWF A1A2A3 and the A1 domain (k_d2_: 1.1 ± 0.5 × 10^–3^ s^-1^ vs k_d2_: 3.2 ± 2.1 × 10^–3^ s^-1^, respectively) (p = 0.31).

Compared to VWF A1A2A3, VWF A3 bound to MMRN1 with a higher K_D_ (representative of lower affinity; K_D_: 2.0 ± 0.4 vs 229 ± 114 × 10^–9^ M, respectively) (p < 0.05; ▶Figure 4D). VWF A3 binding to MMRN1 had slow on-rates (k_a_: 8.8 ± 2.1 × 10^3^ M^-1^s^-1^) and slow off-rates (k_d_: 1.8 ± 0.5 × 10^–3^ s^-1^).

### The effect of VWF A1A2A3 on platelet adhesion to MMRN1 at high shear (1500 s^-1^)

**Figure 5:**
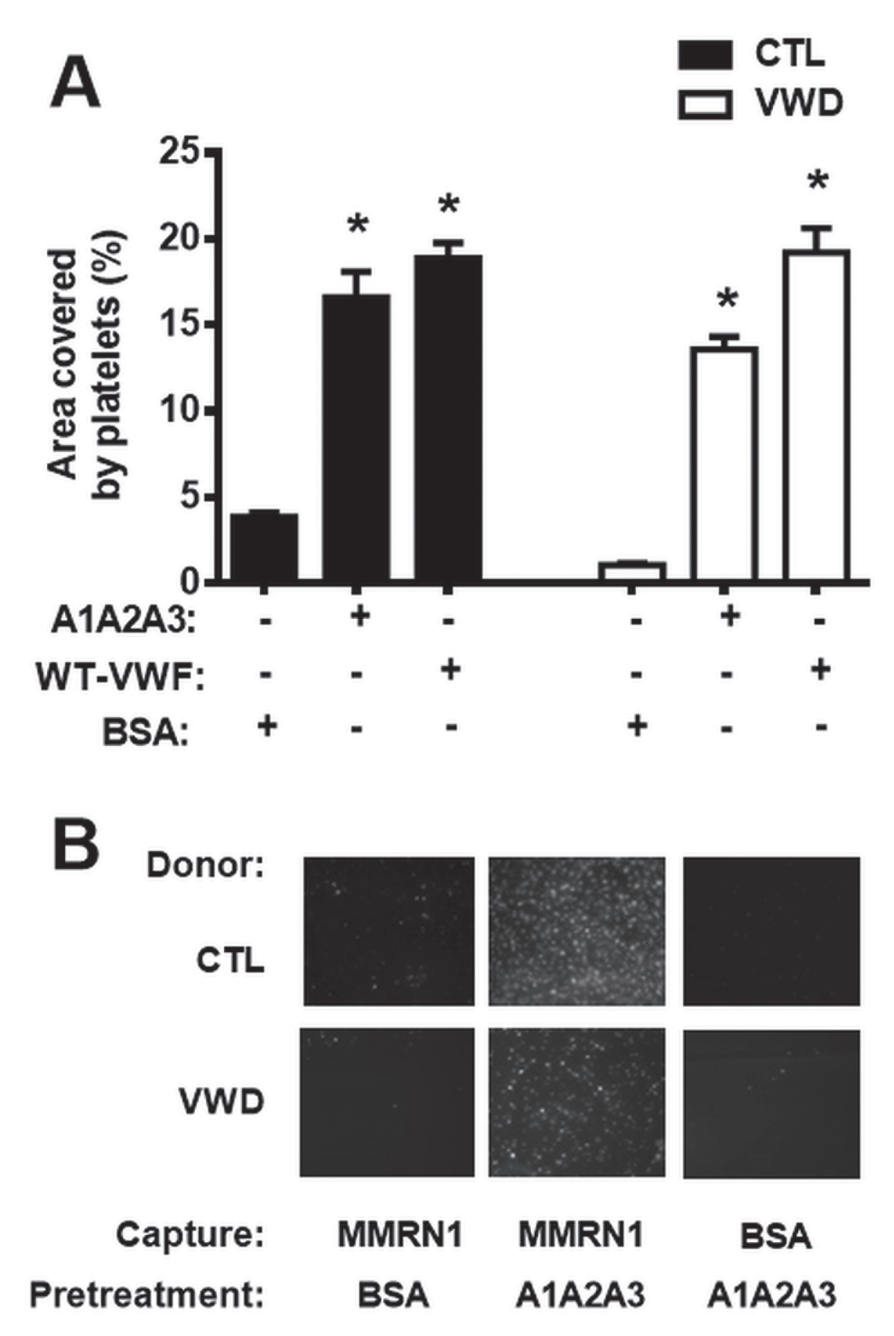
**Effect of VWF A1A2A3 on platelet adhesion to MMRN1, under high shear (1500 s^-1^).** The adhesion (expressed as mean ± SEM for the percentage, %, area covered by platelets) of control (CTL) and type 3 VWD platelets (300 × 10^6^/ml) to immobilised MMRN1, tested at a shear rate of 1500 s^-1^ after pre-treatment with! VWF A1A2A3 (40 nM), WT-VWF (40 nM; positive control) or BSA (negative control). A) Pre-treatments that increased platelet adhesion to MMRN1 are indicated (*; p < 0.001, relative to MMRN1 pre-treated with BSA; data representative of three independent experiments). B) Microscope images (representative of three independent experiments) comparing the adhesion of control (CTL) and type 3 VWD platelets to MMRN1 and BSA-coated surfaces that were pre-treated with VWF A1A2A3 (40 nM) or BSA.

MMRN1 surfaces pre-treated with WT-VWF or VWF A1A2A3 (40 nM) supported the adhesion of resting platelets from controls and a type 3 VWD subject better than MMRN1 surfaces pretreated with BSA (p < 0.001, ▶Figure 5). Control BSA surfaces, similarly pre-treated with VWF A1A2A3, supported ≤ 5 % of the platelet adhesion observed with MMRN1 surfaces (▶Figure 5B).

## Discussion

Roles are emerging for MMRN1 in supporting VWF-dependent platelet adhesion at high shear, a process that requires VWF to associate with MMRN1 to support GPIbα-dependent platelet adhesion (10). The goal of this study was to define the structural features of VWF that support MMRN1 binding and platelet adhesion to MMRN1 at high shear, and determine the affinities of VWF-MMRN1 binding using VWF A domain mutant proteins. We tested the hypothesis that VWF-MMRN1 binding, which is enhanced by exposing VWF to high shear rates (▶ Figure 1A), involves the shear-sensitive A1A2A3 domains of VWF (▶ Figure 1B and ▶ Figure 4). We identified the A1 and A3 domains of VWF as specifically important for VWF binding to MMRN1 (▶ Figure 2 and ▶ Figure 4). VWF A2 domain did not bind to MMRN1 (▶ Figure 2 and ▶ Figure 4A). The entire VWF A1A2A3 region showed higher affinity for MMRN1 (K_D_: 2.0 ± 0.4 × 10^–9^ M) than the individual VWF A1 (K_D_: 39.3 ± 7.7 × 10^–9^ M) and A3 domains (K_D_: 229 ± 114 × 10^–9^ M). Additionally, like WT-VWF exposed to high shear or ristocetin (▶ Figure 1A), VWF A1A2A3 bound to MMRN1 and resting platelets at high shear, and supported high shear platelet adhesion to MMRN1 (▶ Figure 1B and ▶ Figure 5). The affinity and nature of VWF-MMRN1 binding suggests that at sites of vessel injury, where MMRN1 is released from activated platelets and endothelium (12, 13, 15, 16, 45), VWF binding to MMRN1 occurs and facilitates GPIbα-dependent platelet adhesion at high shear (10). Our study extends the known functions of the VWF A domains to include MMRN1 binding.

Shear-induced activation of VWF (or VWF activation by the agonist ristocetin (31)) reveals the cryptic GPIbα binding site in VWF A1 domain (6, 29); similarly, shear and ristocetin revealed MMRN1 binding sites in VWF (▶ Figure 1A). VWF A1A2A3 domain mutant protein bound to MMRN1 without shear or ristocetin, possibly because cryptic binding sites are more exposed in this mutant protein than in the full-length molecule. Our observation that the VWF A1 domain is important for MMRN1 binding suggests that there is potential overlap in the binding sites of GPIbα and MMRN1 within VWF A1 domain (▶ Figures 2-4). However, MMRN1 likely does not impair GPIbα binding to VWF, as A1A2A3-loaded MMRN1 matrices supported platelet adhesion at a high shear rate (▶ Figure 5), demonstrating that VWF A1A2A3 bound to MMRN1 retains its ability to bind to platelet GPIbα. MMRN1 has not been shown to bind GPIbα directly (10), and the observations that MCA4683 (inhibitor of VWF-GPIbα binding) completely inhibited platelet adhesion to MMRN1 at high shear (▶ Figure 3), further emphasises that VWF-GPIbα binding is essential for platelet adhesion to MMRN1 at high shear. MMRN1 binding to VWF A1 domain parallels the observations made on VWF A domain binding to subendothelial collagens (23–27). MMRN1 is a normal constituent of the vascular extracellular matrix (16), and it may enhance VWF binding at sites of vascular damage, like laminin and fibronectin, which also bind VWF and support GPIbα-dependent platelet adhesion (46, 47).

VWF A3 domain, which is structurally homologous to VWF A1 domain, was also important for MMRN1 binding (▶ Figure 2). RU5 and type III collagen peptides that specifically bind to VWF A3 domain, and inhibit collagen binding (43), partially inhibited the binding of VWF to MMRN1 (▶ Figure 2C-D). This suggests that there may be overlap in the binding sites for type III collagen and MMRN1 within VWF A3 domain. Overlap in VWF A3 binding sites for matrix ligands, such as MMRN1 and type III collagen, might provide a redundant mechanism to optimise the capture of VWF at sites of vessel injury, as also postulated for laminin and fibronectin (46, 47).

In the current study, we used VWF A1A2A3 domains to assess the mechanism and affinity of VWF-MMRN1 binding, as previously we found inconsistent binding of WT-VWF to MMRN1 by SPR (10). Furthermore, the highly polymeric nature of both VWF and MMRN1 challenges the estimation of affinity because the combined strength of multiple bonds (instead of a single bond) results in measurements of avidity. We were successful in obtaining affinity estimates using monomeric VWF A1A2A3 (▶ Table 1). VWF A1A2A3 bound to MMRN1 with high, physiologically relevant affinity (K_D_: 2.0 ± 0.4 × 10^–9^ M), that is well below VWF plasma levels (~40 × 10^–9^ M), unlike the modest binding of individual VWF A1 and A3 domains (K_D_: 39.3 ± 7.7 × 10^–9^ M and 229 ± 114 × 10^-9^ M, respectively). Interestingly, the binding of A1A2A3 domain, and the A1 domain, to MMRN1 showed best fit with a two-step, conformational change model, which we reported for factor V, but not factor Va binding to MMRN1(20). It is possible that in the case of VWF binding to MMRN1, the change in conformation might be in the VWF A domains, MMRN1 or both. Our estimate of the affinity of VWF A1A2A3 for MMRN1 suggests that at sites of arterial vascular injury, where VWF undergoes shear-induced activation (1), released MMRN1 binds to plasma VWF to further support platelet adhesion. The data that we obtained by testing individual A domains of VWF, inhibitory antibodies and deletion mutant proteins suggest that multiple VWF A domains contribute to MMRN1 binding, with the main binding site for MMRN1 located within the VWF A1 domain. However, we cannot exclude the possibility that the low binding affinity that was seen with the VWF A3 domain was an artefact of an altered conformation induced by absence of the neighbouring domains. It is also possible that the multimeric structure of WT-VWF enhances the binding of VWF A3 domain to MMRN1, as it influences VWF A3 binding to collagen (39, 48). VWF is known to self-associate at high concentrations (> 500 nM) (49), and this is mediated by interactions with VWF A1 and A2 domains, as well as neighbouring N- and C- terminal domains, but does not require the A3 domain (2, 50). Based on the concentrations of VWF mutant proteins that we tested, we suspect that there was minimal interference from self-association of mutant proteins in our kinetic estimations, particularly for A1A2A3. It is important to note that VWF A domain mutant proteins typically underestimate WTVWF binding affinities by several orders of magnitude because neighbouring functional domains alter VWF binding affinities and avidities (37, 43). Similarly, we likely underestimated the affinity of WT-VWF for MMRN1 by studying the binding of VWF A1A2A3 to MMRN1.

VWD is a heterogeneous disease characterised by defects in VWF quantity (types 1 and 3) and quality/functionality (type 2). Type 2M VWD is the disease subgroup defined by several mutations within VWF A1A2A3 domains that abrogate VWF A1 binding to GPIbα, and the binding of the VWF A1 and A3 domains to collagens (1, 27, 48). The data presented here describes MMRN1 as a novel ligand for VWF A1A2A3 region, and suggests that impaired VWF binding to MMRN1 could occur in some forms of VWD, reducing VWF-GPIbα-dependent platelet adhesion at sites of vessel injury.

**What is known about this topic?**

- Activation of endothelial cells and platelets lead to MMRN1 release from intracellular stores, and MMRN1 binding to outer cell surfaces.
- VWF associates with MMRN1 matrices at high shear rates.
- MMRN1 supports platelet adhesion at high shear by a VWFdependent mechanism involving GPIbα not p_3_ integrins.

**What does this paper add?**

- VWF-MMRN1 binding is enhanced by exposing VWF to shear or treating VWF with ristocetin.
- MMRN1 binds to VWF A1A2A3, and to the individual VWF A1 and A3 domains.
- VWF A1A2A3 binds to MMRN1 with a physiologically relevant binding affinity, whereas the individual VWF A1 and A3 domains bind to MMRN1 with low to modest affinity.
- VWF A1A2A3 binds to MMRN1 and this region of VWF is sufficient to support the GPIbα-dependent adhesion of resting platelets to MMRN1, at a high shear rate.

Our study provides new information on the molecular basis of MMRN1 binding to VWF, which supports platelet adhesion at high shear (10). We propose that at sites of vessel injury, where MMRN1 is released from the storage granules of platelets and endothelial cells (12, 13, 15, 16, 45), MMRN1 binds to VWF A1A2A3 region and other components of the extracellular matrix (including collagen), to support platelet adhesion at arterial shear rates (10, 16, 21). Gene knockout animal models provide important insights on many protein functions (14), so we are now characterizing multimerin 1 functions *in vivo* using mice with a selective deficiency.

## Figures and Tables

**Table 1:** Binding kinetic estimations for the real-time binding between VWF A1A2A3, A1, and A3 binding to MMRN1, as measured by SPR.

VWF analyte	Binding model	Estimated affinity	Estimated association rate constant	Estimated dissociation rate constant
A1A2A3	2-State	K_D_: 2.0 ± 0.4 × 10^–9^ M	k_a1_: 2.6 ± 0.9 × 10^6^ M^-1^s^-1^ k_a2_: 4.2 ± 0.3 × 10^–3^ s^-1^	k_d1_: 2.7 ± 0.3 × 10^–2^ s^-1^ k_d2_: 1.1 ± 0.5 × 10^–3^ s^-1^
A1	2-State	K_D_: 39.3 ± 7.7 × 10^–9^ M	k_a1_: 1.2 ± 0.8 × 10^6^ M^-1^s^-1^ k_a2_: 1.0 ± 0.5 × 10^–2^ s^-1^	k_d1_: 1.3 ± 0.2 × 10^–1^ s^-1^ k_d2_: 3.2 ± 2.1 × 10^–3^ s^-1^
A3	1:1 Langmuir	K_D_: 229 ± 114 × 10^–9^ M	k_a_: 8.8 ± 2.1 × 10^3^ M^-1^s^-1^	k_d_: 1.8 ± 0.5 × 10^–3^ s^-1^
